# The effect of diets with different inflammatory scores on inflammation and sleep in obese subjects: a randomized controlled trial

**DOI:** 10.1590/1806-9282.20240588

**Published:** 2024-09-30

**Authors:** Hakan Toğuç, Hande Öngün Yılmaz, Bülent Yaprak

**Affiliations:** 1 Inonu University, Faculty of Health Sciences, Department of Nutrition and Dietetics – Malatya, Turkey.; 2 Bandırma Onyedi Eylül University, Faculty of Health Sciences, Department of Nutrition and Dietetics – Balıkesir, Turkey.; 3 Malatya Training and Research Hospital, Department of Internal Medicine – Malatya, Turkey.

**Keywords:** Diet, Inflammation, Sleep quality, Obesity, Nutrition

## Abstract

**OBJECTIVE:**

The aim of this study was to investigate the effect of different dietary inflammatory index diets on inflammatory markers, anthropometric measurements, and sleep quality in obese subjects.

**METHODS:**

This study was conducted in a public hospital in Turkey between November 2021 and May 2022. Participants with pro-inflammatory dietary habits were included in the study. Randomly divided into two groups of 33 participants, they were subjected to an anti-inflammatory diet or a control diet for 8 weeks. The study evaluated the anthropometric parameters, inflammatory biomarkers, and sleep quality indices of the diet groups.

**RESULTS:**

Significant reductions in body mass index were observed in both groups, more marked in the anti-inflammatory diet cohort. C-reactive protein levels, indicative of inflammation, also decreased substantially in both groups, with a more marked reduction in the anti-inflammatory diet cohort. Despite the improvement in sleep quality in both groups, the variation was not statistically significant.

**CONCLUSION:**

This study demonstrates the importance of anti-inflammatory diets in nutritional strategies for obesity by reducing body mass index and inflammation.

## INTRODUCTION

Obesity, which is a serious metabolic disorder, arises from a chronic imbalance where calorie intake consistently exceeds energy expenditure, leading to excessive fat accumulation. Globally, obesity rates have doubled in the last four decades, cutting across demographic and socio-economic lines^1,2^. This surge necessitates advanced strategies for its analysis, management, and treatment^3^.

Central to obesity’s pathophysiology is adipose tissue, which significantly influences pathological and physiological processes, including inflammation and immune responses^4^. This tissue’s immune cells secrete both pro- and anti-inflammatory cytokines. While anti-inflammatory cytokines attempt to maintain insulin sensitivity, their pro-inflammatory counterparts promote insulin resistance and trigger adverse lipid metabolism in peripheral tissues, contributing to systemic low-grade inflammation^5^. Additionally, certain adipocyte-secreted molecules intensify chronic inflammation, emphasizing obesity’s characterization as an inflammatory condition^6^.

Furthermore, research indicates a pivotal role for sleep in obesity’s multifaceted etiology. Reduced sleep duration has been associated with hormonal imbalances, affecting appetite regulation, promoting weight gain, and increasing diabetes risk^7^. Insufficient sleep may also encourage excessive food intake, thereby increasing obesity susceptibility and influencing the response to dietary interventions^8^.

Recognizing the need for a comprehensive measure of the effect of diet on inflammation, Cavichia et al. and later Shivappa et al. introduced the dietary inflammatory index (DII), which consists of 45 food and nutrients^9,10^.

The objective of this study was to elucidate the influence of diets characterized by disparate DII scores on inflammatory biomarkers, anthropometric indices, and sleep quality among obese subjects consuming a pro-inflammatory diet.

## METHODS

### Study population, sampling, and ethics

This single-blind, randomized controlled trial was conducted between November 2021 and May 2022 in a public hospital in Turkey. The study included voluntary obese participants who met the inclusion criteria. The sample size was set to a minimum of 28, 14 per group, with 90% power and a 5% margin of error, based on previous DII score data from Shin et al. The study was completed by 33 participants, 16 in the AID group, and 17 in the CD group^11^.

Inclusion criteria were adults aged 19–65 years with a body mass index (BMI) of 30–40 kg/m^2^, no chronic disease, no recent use of special diets or supplements, and no recent inflammatory or serious psychiatric conditions.

The study received ethical approval from the Turgut Özal University Non-Interventional Clinical Research Ethics Committee on November 29, 2021, in accordance with the Declaration of Helsinki (Ref. No: 2021/21). Participants were informed about the study and signed a voluntary consent form.

### Data collection tools and procedures

Socio-demographic data, dietary habits, and sleep quality were assessed using a detailed data collection form, the Pittsburgh Sleep Quality Index (PSQI), and direct interviews. A 3-day dietary record was kept by participants, including one weekend day, to calculate DII scores.

Sleep quality was evaluated using the PSQI, which measures sleep quality over the previous month. A score above 5 indicates poor sleep quality, suggesting a need for clinical evaluation^12,13^.

Height was measured according to the Frankfort horizontal plane criteria, and BMI was calculated using the [weight(kg)/height(m)]^2^ formula. Waist and hip circumferences were measured using non-elastic bands.

Body composition was analyzed using the TANITA BC-730 bioelectrical impedance analysis scale under controlled conditions, including abstaining from vigorous exercise, alcohol, and caffeine and adjusting food and water intake^14^.

Fasting blood samples were analyzed for glucose, triglycerides, total cholesterol, low-density lipoproteins (LDL)-cholesterol, C-reactive protein (CRP), and leukocyte counts. LDL cholesterol was calculated using the Friedewald formula, and measurements were taken at baseline, 4 weeks, and 8 weeks^15^.

### Dietary inflammatory index calculation

The DII is an analytical tool designed by Shivappa et al. to assess the inflammatory potential of diets. To calculate the DII, z-scores are derived from the consumed amounts of various nutrients, reflecting their inflammatory effects. The averages and standard deviations for 45 nutrients were established using data from national nutrition surveys. For this study, the calculation included 32 nutrients due to database limitations. The z-scores were transformed into percentile scores to achieve symmetry, which were then doubled and reduced by one to adjust the distribution. Each nutrient’s score was then multiplied by its specific effect score to compute the cumulative DII, representing the diet’s inflammatory potential.

### Study design

Of the 124 interested participants, 17 were excluded due to negative DII scores. Of the 107 participants with positive DII scores, 48 dropped out, and the remaining 59 were randomly assigned to the anti-inflammatory diet (AID) group or the control diet (CD) group in a single-blind setup, where participants were unaware of their group assignment to prevent bias in their responses and behaviors. For 8 weeks, the AID group followed an anti-inflammatory diet, while the CD group followed a neutral diet. Follow-up assessments at weeks four and eight included PSQI interviews, 3-day diet diaries, and anthropometric measurements. Biochemical parameters were obtained from medical records. The study ended with 16 participants in the AID group and 17 participants in the CD group completing the protocol.

### Implemented diets

Two diet regimens, AID and CD, were formulated based on the DII and adhered to the Turkish Dietary Guidelines-2016 (TÜBER-2016)^16^. These guidelines recommend a macronutrient distribution of 15% protein, 55% carbohydrates, and 30% fat, with an inclusion of one to two servings of fish weekly. Caloric intakes were set to promote moderate weight reduction, with 1,700 kcal/day for females and 1,900 kcal/day for males, targeting moderately inactive individuals aged 19–65.

Anti-inflammatory diet: This diet followed the Mediterranean dietary pattern and TÜBER-2016, providing iso-caloric plans designed to achieve negative DII scores of -3.38 for females and -3.53 for males.

Control diet: The CD was a neutral diet with minimal inflammatory potential, reflected in DII scores of 0.11 for females and 0.03 for males.

Participants underwent face-to-face interviews in the fourth and eighth weeks. During these sessions, dietary records and anthropometric data were collected. Routine biochemical markers were obtained from medical records.

### Statistical analysis

Data were analyzed using IBM SPSS version 22.0 and dietary records through BeBIS 8.2. Descriptive statistics (mean, standard deviation, frequency, and percentage) and distribution normality (histograms and Shapiro-Wilk test) were assessed. Categorical data were analyzed using Pearson’s chi-square and Fisher’s exact tests. Group differences were examined with an independent sample t-test after Levene’s test for equality of variances. A transformation of variables like CRP, sleep quality, neutrophil counts, and BMI was performed to meet parametric test assumptions. Within-group comparisons utilized a one-way analysis of variance (ANOVA) from general linear models (GLM) with repeated measures, while between-group differences were analyzed using a two-way ANOVA from GLM with repeated measures. Post-hoc analyses were conducted using the Bonferroni method, with statistical significance set at p<0.05.

## RESULTS

Comparative analysis of the characteristics of the participants categorized according to dietary groups in Table 1 showed that there was no statistically significant difference between the AID and CD cohorts in terms of age, gender, educational status, employment status, or marital status (p>0.05).

**Table 1:** General characteristics of participants according to dietary groups.

General features	AID Group		CD Group		Test	p
	Mean	SD	Mean	SD		
Age (years)	39.19	8.89	43.65	9.08	t: -1.424	0.164
**Gender**	**n**	**%**	**n**	**%**		
Female	10	30.30	10	30.30	χ^2^=0.047	0.829
Male	6	18.20	7	21.20		
Education status						
Illiterate	3	90.10	2	6.10	*χ^2^=7.198	0.193
Literate	3	90.10	0	0		
Primary school	5	15.20	8	24.20		
Middle school	1	3.00	2	6.10		
High school	0	0	3	9.10		
University	4	12.10	2	6.10		
Occupational status						
Not working	4	12.10	1	3.00	*χ^2^=3.578	0.658
Officer	1	3.00	2	6.10		
Worker	2	6.10	2	6.10		
Self-employment	0	0	1	3.00		
Retired	8	24.20	9	27.30		
Housewife	1	3.00	2	6.10		
Marital status						
Married	15	45.40	15	45.40	χ^2^=0.309	0.582
Single	1	3.00	2	6.10		
Total	16	48.50	17	51.50		

AID: anti-inflammatory diet; CD: control diet; t: independent sample t-test statistic; χ^2^: chi-square test statistic;

*χ^2^: Fisher’s exact test, p<0.05.

Comparison of anthropometric measurements of the participants categorized according to diet group in Table 2 showed a significant decrease in body weight, BMI, body fat percentage, skeletal muscle mass, waist, and hip circumferences (p<0.05) and an increase in body water percentage (p<0.05) in the AID group. Similarly, the CD group showed a significant decrease in body weight, BMI, body fat percentage, muscle mass, waist, and hip circumferences (p<0.05) and an increase in body water percentage (p<0.05). Intergroup comparisons between the AID and CD cohorts revealed significantly greater reductions in body weight and BMI for AID participants (p<0.05). In the AID and CD cohorts, within-group assessments showed a decrease in sleep quality scores (p<0.05), but no significant difference was detected in between-group comparisons.

**Table 2:** Comparison of anthropometric measurements and sleep quality according to groups.

Anthropometric measurements	Times	AID Group		CD Group		Test F2	P^2^
		Mean	SD	Mean	SD		
Body weight (kg)	Beginning	99.79	11.15^a^	100.51	15.52^a^	3.756	**0.029**
	1. Control	94.79	11.14^b^	96.74	15.11^b^		
	2. Control	91.19	11.38^c^	93.42	14.81^c^		
	Test F1	197.936		162.575			
	p^1^	**<0.001**		**<0.001**			
Body mass index (kg/m^2^)*	Beginning	37.60	2.83^a^	36.61	2.979^a^	3.999	**0.033**
	1. Control	35.72	2.97^b^	35.24	3.08^b^		
	2. Control	34.36	3.20^c^	34.02	3.01^c^		
	Test F1	152.452		167.186			
	p^1^	**<0.001**		**<0.001**			
Body fat percentage (%)	Beginning	44.72	2.71^a^	44.21	4.81^a^	2.752	0.072
	1. Control	43.07	2.61^b^	43.11	5.25^a^		
	2. Control	40.61	3.34^c^	41.41	5.28^b^		
	Test F1	68.504		25.687			
	p^1^	**<0.001**		**<0.001**			
Body muscle weight (kg)	Beginning	52.02	4.04^a^	51.78	4.68^a^	0.071	0.873
	1. Control	50.70	4.27^b^	50.33	4.59^b^		
	2. Control	49.72	4.46^ab^	49.14	4.48^c^		
	Test F1	4.228		19.226			
	p^1^	**0.046**		**<0.001**			
Body water content (%)	Beginning	40.29	2.33^a^	40.82	4.06^a^	0.440	0.646
	1. Control	41.34	1.93^a^	41.73	4.68^a^		
	2. Control	43.00	2.66^b^	43.05	4.75^b^		
	Test F1	30.616		16.227			
	p^1^	**<0.001**		**<0.001**			
Waist circumference (cm)	Beginning	110.62	6.76^a^	109.00±	7.10^a^	0.933	0.355
	1. Control	107.37	6.54^b^	105.41	6.86^b^		
	2. Control	106.56	7.89^b^	103.71	7.74^c^		
	Test F1	13.515		68.815			
	p^1^	**0.002**		**<0.001**			
Hip circumference (cm)	Beginning	117.13	11.71^a^	116.35	11.32^a^	0.586	0.493
	1. Control	114.12	11.11^b^	113.06	10.54^b^		
	2. Control	112.12	10.66^c^	110.65	10.23^c^		
	Test F1	61.622		73.901			
	p^1^	**<0.001**		**<0.001**			
Sleep quality score**	Beginning	8.13	3.03^a^	5,82	2,13^a^	3.121	0.063
	1. Control	5.1	1.28^b^	5.12	2.71^a^		
	2. Control	2.44	1.46^c^	2.88	1.73^b^		
	Test F1	29.963		16.948			
	p^1^	**<0.001**		**<0.001**			

p1: intra-group measurement comparison; p2: inter-group measurement comparison; AID: anti-inflammatory diet; CD: control diet.

*Logarithmic transformation was applied in base 10;

**square root transformation applied. Different letters in the same row indicate difference; F1: analysis of variance for one-way repeated measures; F2: analysis of variance for two-way repeated measures, p<0.05.

Statistically siginificant values are indicated in bold.

In the comparison made in Table 3 regarding the evaluation of the biochemical measurements of the participants during the experiment, in the AID group, within-group analyses showed significant decreases in fasting blood glucose, serum triglycerides, LDL cholesterol, total cholesterol, and CRP concentrations (p<0.05), while leukocytes, lymphocytes, and neutrophils remained stable (p>0.05). In contrast, the CD group showed no significant changes in any of the biochemical parameters evaluated, including fasting blood glucose, serum triglycerides, LDL cholesterol, total cholesterol, CRP, leukocytes, lymphocytes, and neutrophils (p>0.05). Intergroup comparison between the AID and CD cohorts revealed a significant reduction in CRP levels in the AID group, indicating a weakened inflammatory response (p<0.05).

**Table 3:** Comparison of biochemical measurements according to groups.

Biochemical measurements	Times	AID Group		CD Group		Test F2	P^2^
		Mean	SD	Mean	SD		
Fasting blood glucose (mg/dL)	Beginning	94.06	5.45^a^	91.41	7.27	1.766	0.179
	1. Control	92.87	4.30^ab^	92.71	4.25		
	2. Control	87.81	8.46^b^	90.35	6.40		
	Test F^1^	5.341		0.787			
	p^1^	**0.010**		0.464			
Triglycerides (mg/dL)*	Beginning	126.00	27.27^a^	123.41	28.67	1.155	0.322
	1. Control	114.29	25.40^ab^	111.85	33.62		
	2. Control	103.08	30.73^b^	112.88	34.15		
	Test F^1^	4.671		2.623			
	p^1^	**0.032**		0.088			
LDL-cholesterol (mg/dL)	Beginning	99.04	17.29^a^	100.42	21.91	1.526	0.225
	1. Control	93.88	18.58^ab^	97.96	21.90		
	2. Control	86.90	16.59^b^	96.65	22.22		
	Test F^1^	0.839		0.775			
	p^1^	**0.013**		0.469			
Total cholesterol (mg/dL)	Beginning	170.12	18.62^a^	173.65	25.92	1.339	0.269
	1. Control	162.09	23.47^ab^	168.47	27.38		
	2. Control	155.03	21.54^b^	166.76	26.72		
	Test F^1^	6.691		2.761			
	p^1^	**0.004**		0.099			
CRP (mg/l)**	Beginning	9.65	6.88^a^	5.03	3.81	9.565	**<0.001**
	1. Control	6.59	6.04^b^	4.25	3.71		
	2. Control	4.14	3.67^b^	4.30	4.16		
	Test F^1^	25.140		1.245			
	p^1^	**<0.001**		0.302			
Leukocytes (10^3^/μl)	Beginning	7.69	2.63	7.01	1.37	0.316	0.680
	1. Control	7.26	1.90	6.91	1.20		
	2. Control	7.67	2.11	6.96	1.40		
	Test F^1^	0.732		0.072			
	p^1^	0.489		0.931			
Lymphocytes (10^3^/μl)	Beginning	2.15	0.71	2.27	0.82	0.177	0.788
	1. Control	2.14	0.48	2.22	0.41		
	2. Control	2.35	0.46	2.34	0.53		
	Test F^1^	1.423		0.240			
	p^1^	0.255		0.788			
Neutrophils (10^3^/μl)***	Beginning	4.64	1.64	3.99	1.01	0.955	0.374
	1. Control	4.21	1.07	4.10	0.94		
	2. Control	4.26	0.99	4.00	0.99		
	Test F^1^	0.405		0.601			
	p^1^	0.627		0.518			

p1: Comparison of within-group measurement changes; p2: comparison of between-group measurement changes; AID: anti-inflammatory diet; CD: control diet..

*Logarithmic transformation was applied at base 10 by adding a constant value;

**square root transformation was applied;

***logarithmic transformation applied at base 10. Different letters indicate difference. F1: analysis of variance for one-way repeated measures; F2: analysis of variance for two-way repeated measures, p<0.05.

Statistically siginificant values are indicated in bold.

## DISCUSSION

Obesity creates significant socioeconomic burdens on individual, social, and health systems^17^. Recent strategies in obesity treatment take into account the role of DII in diet planning. This study divided the participants into two groups: one group followed AIDs, while the other group (CD) followed a mixed regime. Both groups showed improvements in body measurements. However, the DII group achieved greater weight and BMI reduction, suggesting an advantage for AIDs in weight loss. However, a study by Babatunde et al. showed a significant reduction in these measures among AID participants compared to the control group^18^. In contrast, Mayr et al. found no significant difference in body weight or fat measures between coronary heart disease patients on AIDs and those on a low-fat diet, suggesting that the effects of AIDs may not apply universally^19^. Similarly, Kenđel Jovanović et al. observed significant reductions in obesity indicators in subjects on both AIDs and standard diets but found no significant difference between the two groups^20^.

The increasing incidence of obesity-related chronic conditions such as diabetes and cardiovascular disease emphasizes the importance of rigorous monitoring of biochemical markers during dietary changes^21^. Mayr et al. investigated the effects of a Mediterranean-style AID compared to a low-fat diet for 6 months and found no significant change in fasting blood glucose between the groups^19^. However, Salama et al. highlighted the potential efficacy of the diet by documenting a marked decrease in these levels following an anti-inflammatory intervention^22^. This research further supports the role of AID in modulating fasting blood glucose, although the results do not contrast sharply with CDs. These observations reveal the potential of AIDs in diabetes management, given the increasing global prevalence of diabetes.

The interaction between obesity, characterized by persistent low-grade inflammation, and increased cardiovascular risk underlines the importance of dietary interventions in modulating lipid profiles and inflammatory markers^19^. Salama et al. observed a significant decrease in markers such as total cholesterol, LDL-cholesterol, and triglycerides among obese women with PCOS following an AID^22^, whereas Turner-McGrievy et al. reported no significant changes in similar cohorts^23^. Interestingly, Mayr et al. recorded a decrease in triglycerides among participants following the Mediterranean diet, a variant of AID, without significant changes in other lipid parameters^19^. In this study, a significant decrease in triglyceride, total cholesterol, and LDL-cholesterol levels was found in the AID-treated group but not in the control group. These results suggest that the AID model may be effective in reducing serum triglyceride, total cholesterol, and LDL cholesterol levels.

The nuanced relationship between diet, inflammation, and various health indices in obesity is increasingly being recognized, emphasizing that the AID has a high potential to influence these parameters, although there are conflicting results in existing research. Turner-McGrievy et al. and Babatunde et al. found no significant changes in CRP levels in AIDs, whereas Salama et al. documented notable reductions suggesting variable responses to dietary interventions^18,22,23^. The present study highlights a significant reduction in CRP levels in participants with AIDs, suggesting the possible role of diet in reducing the systemic inflammation that accompanies obesity. These data support the idea that diets specifically targeting inflammatory pathways may significantly influence the management of inflammatory aspects of obesity and potentially lead to new therapeutic directions^19,23^.

In addition, emerging evidence links inflammatory diets with impaired sleep quality. Studies among different populations have found that poorer sleep is associated with higher DII scores, extending the influence of dietary patterns beyond traditional metabolic parameters^24,25^. Similarly, Günal observed that among 3,072 women, poor sleep quality was correlated with elevated energy and macronutrient consumption^26^. In the present study, improvements in sleep quality were noted in both the AID and CD groups, with no significant difference between the groups, suggesting that factors other than diet type may contribute to sleep outcomes. However, the fact that sleep tended to improve in the AID group points to the potential adjunctive role of diet in managing sleep disturbances in an inflammatory background. Collectively, these insights argue for an integrated approach to obesity research where dietary interventions are examined through a multidimensional lens encompassing systemic inflammation, immune function, and sleep quality.

## CONCLUSION

These findings support the use of AIDs to manage inflammation and sleep problems in obese populations, but further studies are needed to fully understand the complex diet-inflammation-metabolism interactions.
